# Regulation of soluble vascular endothelial growth factor receptor (sFlt-1/sVEGFR-1) expression and release in endothelial cells by human follicular fluid and granulosa cells

**DOI:** 10.1186/1477-7827-3-57

**Published:** 2005-10-25

**Authors:** Ruth Gruemmer, Karin Motejlek, Daniela Berghaus, Herbert A Weich, Joseph Neulen

**Affiliations:** 1 Institute of Anatomy, University Hospital, Essen, Germany; 2 Clinic of Gynecological Endocrinology and Reproductive Medicine, RWTH Aachen, Germany; 3 Dep. Gene Regulation and Differentiation, GBF Braunschweig, Germany

## Abstract

**Background:**

During the female reproductive cycle, follicular development and corpus luteum formation crucially depend on the fast generation of new blood vessels. The importance of granulosa cells and follicular fluid in controlling this angiogenesis is still not completely understood. Vascular endothelial growth factor (VEGF) produced by granulosa cells and secreted into the follicular fluid plays an essential role in this process. On the other hand, soluble VEGF receptor-1 (sFlt-1) produced by endothelial cells acts as a negative modulator for the bioavailability of VEGF. However, the regulation of sFlt-1 production remains to be determined.

**Methods:**

We analyzed the influence of human follicular fluid obtained from FSH-stimulated women as well as of human granulosa cell conditioned medium on sFlt-1 production in and release from human umbilical vein endothelial cells (HUVEC) in vitro. Soluble Flt-1 gene expression was determined by RT-PCR analysis, amount of sFlt-1-protein was quantified by Sandwich-ELISA.

**Results:**

Human follicular fluid as well as granulosa cell-conditioned medium significantly inhibit the production of sFlt-1 by endothelial cells on a posttranscriptional level. Treatment of cultured granulosa cells with either hCG or FSH had not impact on the production of sFlt-1 inhibiting factors. We further present data suggesting that this as yet unknown sFlt-1 regulating factor secreted by granulosa cells is not heat-sensitive, not steroidal, and it is of low molecular mass (< 1000 Da).

**Conclusion:**

We provide strong support that follicular fluid and granulosa cells control VEGF availability by down regulation of the soluble antagonist sFlt-1 leading to an increase of free, bioactive VEGF for maximal induction of vessel growth in the ovary.

## Background

Angiogenesis is a rare process in normal adult organs predominantly occurring during wound healing and tumor growth. However, under physiological conditions it plays an important role in the female reproductive tract with regard to follicular development, corpus luteum formation, and uterine endometrial proliferation during the menstrual cycle [1,2]. Here, the cyclic corpus luteum of the ovary is the organ with the strongest physiological angiogenesis [3,4]. Defects in ovarian angiogenesis may contribute to a variety of disorders including anovulation and infertility, pregnancy loss, ovarian hyperstimulation syndrome, and ovarian neoplasms [5-7].

During follicular growth, angiogenesis is restricted to the theca cell layer. After ovulation, however, massive angiogenesis occurs and new blood vessels penetrate the basement membrane of the follicle invading the growing corpus luteum [8]. The establishment of such a complex capillary network requires precise timing. Angiogenesis depends on a balance between positive and negative endothelial regulators [9]. Among the many endothelial regulators, vascular endothelial growth factor (VEGF) has been characterized as the most potent promoter of angiogenesis. This key regulator acts specifically on endothelial cells by stimulating cell growth, differentiation, migration and permeability [10,11]. VEGF, especially the isoforms VEGF-A_121 _and VEGF-A_165_, are produced by human granulosa cells [5,12-15], and VEGF-dependent angiogenesis is essential for corpus luteum development [16]. VEGF expression in granulosa cells can be increased by gonadotropins (FSH, hCG) [17,18]. The biological activity of VEGF is mediated by two tyrosine kinase family receptors that are located on endothelial cells (VEGFR-1 = flt-1, VEGFR-2 = KDR) [19]. Binding of VEGF to either of the receptors induces autophosphorylation and signal transduction. Besides these transmembrane receptors, a soluble receptor (sFlt-1) is generated in endothelial cells by differential splicing of the VEGFR-1 mRNA [20,21]. Soluble Flt-1 retains full VEGF binding potency and acts as an inhibitor of VEGF bioactivity by sequestering VEGF, thus reducing the ligand binding to transmembrane and signalling receptors [22,23]. It plays a pivotal role in the generation of vascular diseases like pre-eclampsia or intra-uterine growth retardation [24], and could be linked to the ovarian response to stimulation protocols [25].

The regulation of sFlt-1 production in endothelial cells remains to be determined. In the ovary, it could be demonstrated in high amounts in follicular fluid aspirated during IVF oocyte retrieval. The function of the avascular granulosa cell layer in controlling angiogenesis in the vicinity of developing follicles is still a matter of discussion. In the present study we analyzed the influence of human follicular fluid as well as of human granulosa cell conditioned medium on Flt-1 production and release by human endothelial cells.

## Materials and methods

### Follicular fluid

Follicular fluid was obtained by follicular aspiration from 15 FSH-treated women (34.6 ± 5.6 years) undergoing oocyte retrieval for in vitro fertilization (IVF/ICSI) at the Department of Gynecological Endocrinology at the University Hospital Aachen, Germany. The experimental design was approved by the local ethical committee of the University Hospital Aachen (# EK 2008). Written informed consent was obtained from patients individually. IVF/ICSI was performed due to tubal occlusion (7), andrological (6) or idiopathic (2) reasons. Ovarian stimulation and oocyte retrieval were performed as described previously [26]. After removal of oocytes, follicular fluids of individual patients were pooled, and centrifuged at 500 g for 5 min. Supernatants were frozen at -20°C until further processing.

Follicular fluid was partially purified by using two successive ultrafiltration units with different molecular weight cut offs (MWCOs). To prevent clogging of filters, a 100000 Da ultra-filter (Millipore GmbH, Eschborn, Germany) was used as a pre-filter. The first flow through was consecutively treated with a 1000 Da filter unit (Pall Life Sciences, Ann Arbor, MI). Depending on the follicular fluid's viscosity centrifugation steps were carried out both times for 4–6 hours at 4400 g and 4°C. The final flow through was then incubated either directly with HUVECs or treated further as follows: heating for 10 minutes at 96°C, or dialysis with 100 Da MWCO membranes (Spectrum Laboratories Inc., Rancho Dominguez, CA) equilibrated against two changes of a two hundredfold volume PBS over a 6 hour period at 4°C. In another experiment the partially purified follicular fluid was stirred with dextran-coated charcoal (Sigma-Aldrich Chemie GmbH, Taufkirchen, Germany) at a concentration of 10 mg/ml for 30 minutes at room temperature to absorb free steroids and fatty acids. Any precipitated material or charcoal was removed by centrifugation for 5 minutes at 13 600 g and 4°C.

### Granulosa cell culture

Human granulosa cells were obtained by follicular aspiration from FSH-treated women as described before [26]. Cells were plated at a density of 5 × 10^5 ^cells/well in 6-well dishes and cultured in M199 Earle's Medium supplemented with 10% FCS, 2 mM L-glutamine, and 1% Pen/Strep (all Biochrom, Berlin, Germany) at 37°C in 95% air-5% CO_2 _humidified environment. In one experimental group, medium was additionally supplemented with FSH (Gonal F, Serono, 100 ng/ml), in another experimental group with hCG (Pregnesin, Serono, 1 IU / ml). Cell culture medium was changed after 24 hours and was then harvested after 4 days of culturing and stored at -20°C until further use.

### Endothelial cell culture

Human umbilical vein endothelial cells (HUVEC) were isolated from umbilical cords and cultured as described previously [26]. For each experiment, HUVECs of 3–5 umbilical veins were pooled and seeded in 6- or 12-well plates. At confluence after two days of culturing cells were incubated either with culture medium alone, with culture medium containing 30% follicular fluid (in those experiments with constant concentration of follicular fluid) or with 30% GC-conditioned medium, respectively. After up to 4 days of incubation at 37°C culture medium was collected, centrifuged and supernatant was frozen at -20°C, endothelial cells were harvested and frozen at -20°C until RNA-preparation. For each experimental design HUVECs were separately incubated with follicular fluid or GC-conditioned medium, respectively, of at least 5 different patients.

### Proliferation studies

Human umbilical vein endothelial cells were seeded into 24-well-plates with a density of 50000 cells per well. Cells were incubated with culture medium containing 30% follicular fluid for up to 4 days or with culture medium alone as a control. Growth rates were determined in nine experiments with follicular fluid of nine different patients using an electronic Coulter counter (CASY 1, Schärfe System, Reutlingen, Germany). Each probe has been measured twice.

### Quantification of sFlt-1 protein

Total sFlt-1 concentration in HUVEC supernatant was quantified with a specific enzyme-linked immunosorbent assay (ELISA) (RELIATech, Braunschweig, Germany [22] and BMS268, Bender MedSystems GmbH, Vienna, Austria). Intra- and inter-assay co-efficiencies were CV<10% and CV<20%, respectively. Soluble Flt-1 ELISA analyses were performed in duplicate for each probe.

### RT-PCR

Isolation of total RNA from eutopic endometrial tissues as well as from endometrial tissue grown in nude mice was performed using the RNeasy Minikit^® ^(Qiagen, Hilden, Germany) according to the manufactures instructions. The concentration of RNA was determined spectrophotometrically and the RNA was stored at -80°C until use. Reverse transcription of RNA from HUVECs treated with follicular fluid or granulosa cell conditioned medium was carried out as described previously [26]. Briefly, two micrograms of total RNA were digested with DNase I (Invitrogen, Karlsruhe, Germany) and transcribed into cDNA by reverse transcription with M-MLV Reverse Transcriptase (Invitrogen, Karlsruhe, Germany) using an oligo (dT)_16 _primer in a total volume of 50 μl. Reverse transcription was performed for 60 min at 37°C in a thermocycler (Biometra, Göttingen, Germany) followed by 10 min at 90°C. 4 μl of the RT-reaction were used for PCR experiments. The following primers were used: sFlt-1 forward 5'-GCACCTTGGTTGTGGCTGAC-3'; sFlt-1 reverse 5'-AATGTTTTACATTACTTTGTGTGG-3' (product size 510 bp), β-actin forward 5'-ACCAACTGGGACGACATGGAGAAAA-3', β-actin reverse 5'-TACGGCCAGAGGCGTACAGGGATAG-3' (product size 214 bp). Amplifications were run in 50 μl volume using BioTherm Taq polymerase (Genecraft, Muenster, Germany) for 35 amplification cycles of 30 sec denaturation at 94°C, 45 sec annealing at 62°C and 30 sec elongation at 72°C. The PCR amplification was followed by a 10 minute final extension at 72°C. The conditions were chosen so that the sflt-1 cDNA as well as the control β-actin cDNA were in the exponential phase of amplification and did not reach a plateau at the end of the amplification protocol. The generated PCR amplification products were electrophoresed on a 2% agarose gel and detected by ethidiumbromide staining. PCR products were normalized to β-actin by densitometric analysis using a Gel imager (Intas, Goettingen, Germany) and were relatively quantified (Gelscan Professional V4.0).

### Statistical analysis

Statistical analysis was performed using the non-parametric Mann-Whitney test. The level of significance was set at p < 0.05.

## Results

### Regulation of endothelial cell proliferation and sFlt-1 secretion by follicular fluid

**Figure 1:**
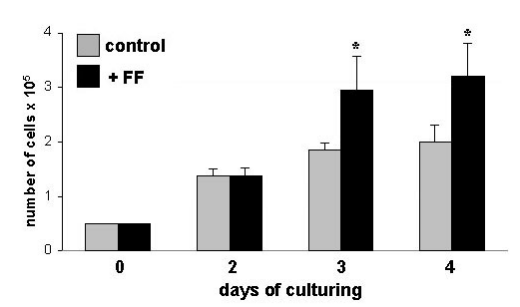
Proliferation of HUVECs incubated with culture medium containing 30% follicular fluid (+FF) or incubated with culture medium alone (control) for up to 4 days. From day 3 onwards a significant increase in cell number can be observed for those endothelial cells treated with follicular fluid compared to controls. * = p < 0.05.

**Figure 2:**
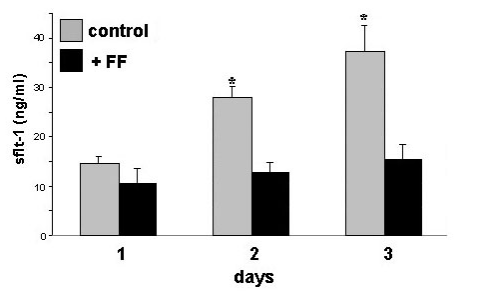
Amount of sFlt-1 in the culture supernatant of HUVECs treated with medium only (grey bars) or with medium containing 30% human follicular fluid (FF, black bars) for up to 3 days. From day 2 onwards a significant increase in sFlt-1 content can be observed for those endothelial cells treated with medium only but not for those incubated with medium containing follicular fluid. * = p < 0.05.

**Figure 3:**
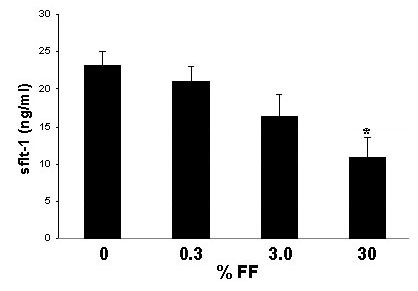
Amount of sFlt-1 in the culture supernatant of HUVECs after 4 days of incubation with medium containing different concentrations of human follicular fluid. Amount of sFlt-1 decreases with increasing concentrations of follicular fluid showing a significant inhibition of sFlt-1 production at a concentration of 30%. * = p < 0.05.

Incubation of HUVECs with culture medium containing 30% follicular fluid led to a significant increase in cell number compared to controls on day 3 and 4 of culturing (Fig. 1). Analyzing secretion of sFlt-1 by endothelial cells, HUVECs secreted 14.63 ± 1.36 ng sFlt-1 per ml into the culture medium during 24 hours of monolayer culture (Fig. 2). After 3 days of culture the amount of secreted sFlt-1 accumulated to 37.2 ± 5.2 ng/ml. This increase in sFlt-1 -1 was inhibited by the presence of 30% follicular fluid in the culture medium (Fig. 2). The inhibition of sFlt-1 production was proven to show a dose-effect as it was dependent on the concentration of follicular fluid in the culture medium. The amount of sFlt-1 decreased with increasing concentration of follicular fluid, showing a significant inhibition of sFlt-1 production at a concentration of 30% (Fig. 3).

**Figure 4:**
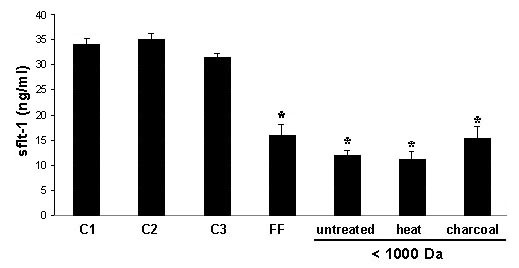
Quantification of sFlt-1 in endothelial cell culture supernatant. Neither heat inactivation of culture medium containing FCS (C2) nor absence of FCS (C3) has a measurable effect on sFlt-1 production of endothelial cells compared to incubation with untreated control medium containing FCS (C1). The significant inhibitory effect of untreated human follicular fluid (FF) on sFlt-1 production is maintained after ultra filtration leaving only molecules smaller than 1000 Dalton. This inhibition is not prevented neither by heat inactivation nor by charcoal treatment of the follicular fluid-flow through. All follicular fluids have been added to the culture medium at a concentration of 30%. * = p < 0.05

To exclude an unspecific role of FCS on sFlt-1 production in HUVECs, culture medium containing FCS had been either untreated, heat inactivated or FCS was omitted. There was no measurable effect of either of these controls on sFlt-1 production of endothelial cells (Fig. 4). To analyze the follicular fluid in regard to factors possibly responsible for sFlt-1 regulation, follicular fluid has been partially purified by ultrafiltration. The resulting flow through containing only molecules smaller than 1000 Dalton still significantly inhibited sFlt-1 secretion by endothelial cells. Moreover, this effect could not be prevented by exposure of the flow through to heat or charcoal treatment (Fig. 4).

### Transcription of sFlt-1 is not regulated by follicular fluid

**Figure 5:**
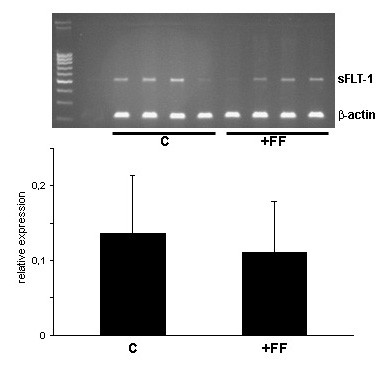
RT-PCR of mRNA of HUVECs incubated with medium only (C) or with medium containing 30% follicular fluid (+FF). Densitometric evaluation revealed no significant difference in mRNA-expression of sFlt-1 between these two experimental groups at p < 0.05.

RT-PCR analysis of HUVECs incubated with medium containing 30% follicular fluid revealed a slight but not significant decrease in sFlt-1 expression compared to endothelial cells treated with medium only (Fig. 5), pointing to a presumable posttranscriptional regulatory mechanism of this soluble receptor by follicular fluid.

### Regulation of sFlt-1 secretion by granulosa cell-conditioned medium

**Figure 6:**
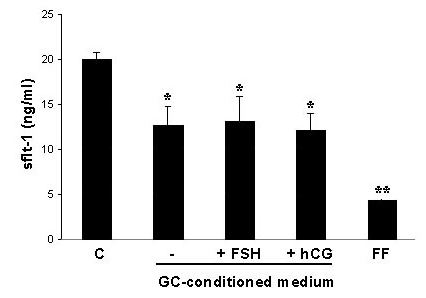
Amount of sFlt-1 in the culture supernatant of HUVECs incubated for 4 days with medium containing 30% granulosa cell (GC)-conditioned medium. Addition of GC-conditioned medium leads to a significant inhibition of sFlt-1 production of HUVECs (*). Treatment of granulosa cells with FCS or hCG during conditioning of medium has no influence on this inhibition. Incubation with follicular fluid (FF) of the same patients leads to a significant reduced production of sFlt-1 compared to controls as well as to treatment with GC-conditioned medium (**). p < 0.05.

This inhibition of sFlt-1 production by endothelial cells was also obtained by incubation of HUVECs with granulosa cell-conditioned medium. At the concentration of 30% granulosa cell-conditioned medium a significant inhibition of sFlt-1 production could be observed after 4 days of culturing (Fig. 6). This inhibition was not influenced by incubation of the granulosa cell culture either with FCS or hCG. In general, however, treatment with granulosa cell-conditioned medium resulted in a lesser inhibition of sFlt-1 production compared to the inhibition by corresponding follicular fluid of the same patients (Fig. 6).

## Discussion

In the present study we demonstrate that human follicular fluid significantly increases proliferation and inhibits the production of the VEGF antagonist sFlt-1 in endothelial cells. It is proven that granulosa cells produce large amounts of VEGF which plays the key role in corpus luteum angiogenesis in vivo [16]. The biological activity of VEGF depends on the availability of this protein to its transmembrane receptors Flt-1 (VEGFR-1) and KDR (VEGFR-2). Soluble Flt-1 is secreted by endothelial cells [20] and acts as a receptor antagonist by sequestering free VEGF, thus repressing angiogenesis mediated by VEGF [22,23]. Soluble Flt-1 may modulate VEGF activity in physiological as well as in patho-physiological angiogenesis in the female reproductive tract. It is secreted by the placenta and released into the maternal circulation during pregnancy [27], and it could be shown that preeclampsia is associated with increased levels of sFlt-1 [24,28-30]. In addition, it could be shown that excess in sFlt-1 goes in parallel with poor response to gonadotropins in stimulation protocols due to the decreased availability of bioactive VEGF [25]. An influence of sFlt-1 on physiological angiogenesis in the ovary was shown by Ferrara and co-workers [16] who reported that treatment with truncated sFlt-1 receptors resulted in complete suppression of corpus luteum angiogenesis in a rat model.

The role of the avascular granulosa cell layer in controlling angiogenesis in the developing follicles is still not clarified. It has been shown before that human follicular fluid contains angiogenic factors such as basic fibroblast growth factors [31], angiogenin [32] and VEGF [33] and that follicular fluid has the capacity to induce angiogenesis [34]. As demonstrated in the present study, the inhibition of sFlt-1 by factors secreted by granulosa cells, thus increasing the amount of free, bioactive VEGF, may represent another regulatory level involved in the angiogenic cascade. Though it is known that soluble receptor protein is generated by alternative splicing of Flt-1 pre-mRNA [35] and exogenous sFlt-1 can dramatically inhibit biological actions of VEGF, there is still little information about physiological functions of sFlt-1 or the mechanisms controlling its biosynthesis. We demonstrated here that factors secreted by granulosa cells and which are contained in follicular fluid inhibit sFlt-1 production, and that this regulation mainly occurs on a posttranscriptional level. This is concordant with our former microarray analyses showing that sFlt-1 mRNA was not amongst the genes significantly regulated by follicular fluid in endothelial cells [26]. A post-transcriptional control of sFlt-1 expression could also be demonstrated by Huckle and Roche [36] using cleavage-polyadenylation mutants of the mouse Flt-1 intron 13. Thus, an opportunity exists for granulosa cells to regulate sFlt-1 protein expression at a widely constant rate of sFlt-1 gene transcription. However, in cyclic human endometrium regulation of sFlt-1 could be demonstrated on a transcriptional level [37] pointing to different regulatory pathways in different organs. The weaker effect of granulosa cell conditioned medium on inhibition of sFlt-1 compared to follicular fluid could be due to a lower concentration of the inhibitory factor in the granulosa cell conditioned medium since this medium was harvested already after 4 days. Though FSH as well as LH / hCG application are able to increase VEGF expression in human granulosa cells [17,18], treatment of cultured luteal granulosa cells either with hCG or FSH had no effect on the inhibition of sFlt-1 secretion. Redmer and co-workers [38] showed that luteal cells secrete a non-steroidal factor which stimulates migration of endothelial cells and that pre-treatment of the luteal cultures with hCG or FSH had no effect on the endothelial cell migration stimulating activity of luteal cell conditioned media. Since the granulosa cells have been exposed to FSH in vivo, a regulatory effect of this hormone can not be excluded. However, depletion of FSH in vitro had no effect on the production of this factor. The nature of the inhibitory factor(s) secreted by granulosa cells is still unknown. As demonstrated here, the factor is of low molecular mass (< 1000 Da) and heat inactivation does not change the inhibitory effect on sFlt-1 secretion. In addition, a steroidal nature can be excluded since this effect is not be eliminated by charcoal treatment.

A complex cascade of events regulated on a transcriptional as well as post-transcriptional level might contribute to the initiation, progression, morphogenesis and regression of blood vessels in the ovary. We have shown previously that after the LH surge, yet before ovulation, granulosa cells secrete factors resulting in destabilization of vessel walls by down-regulating of fibulin-5 and elastin and up-regulation of angiopoietin-2 [26]. In addition, granulosa cells may improve the sprouting of new vessels by down-regulation of the soluble antagonist sFlt-1 thus enhancing the amount of bioactive VEGF. This suppression of sFlt-1 immediately before corpus luteum formation may act to rapidly promote and fine tune angiogenesis in this ephemeral organ. The release in the ovary could be one factor which helps to explain the temporal and spatial discrepancy between the high expression of VEGF in the granulosa cells and the restriction of angiogenesis to the thecal layer in the pre-ovulatory follicle. Disruption of this balance between VEGF and sFlt-1 may result in a disturbed physiological state or various pathological conditions. Understanding the underlying molecular mechanisms which regulate the complex angiogenic cascade is a major challenge with implications for the understanding of blood vessel growth and regression in reproductive biology as well as in pathological conditions.

## Authors' contributions

RG participated in the design of the study, carried out cell culture experiments, performed RT-PCR, performed the statistical analysis and drafted the manuscript. KM and DB carried out part of the cell culture experiments. HAW carried out protein quantification by ELISA. HAW and JN contributed to the conception and design of the study and revised the draft critically. All authors read and approved the final manuscript.
